# Supplementary material for: Influenza in Liver and Kidney Transplant Recipients: Incidence and Outcomes
Source: Microbiol Spectr. 2023 Mar 28;11(2):e03226-22. doi: 10.1128/spectrum.03226-22 (PMC10101112; doi:10.1128/spectrum.03226-22)
Supplement: Supplemental file 1 — Supplemental material. Download spectrum.03226-22-s0001.pdf, PDF file, 0.1 MB [file spectrum.03226-22-s0001.pdf]

Supplementary

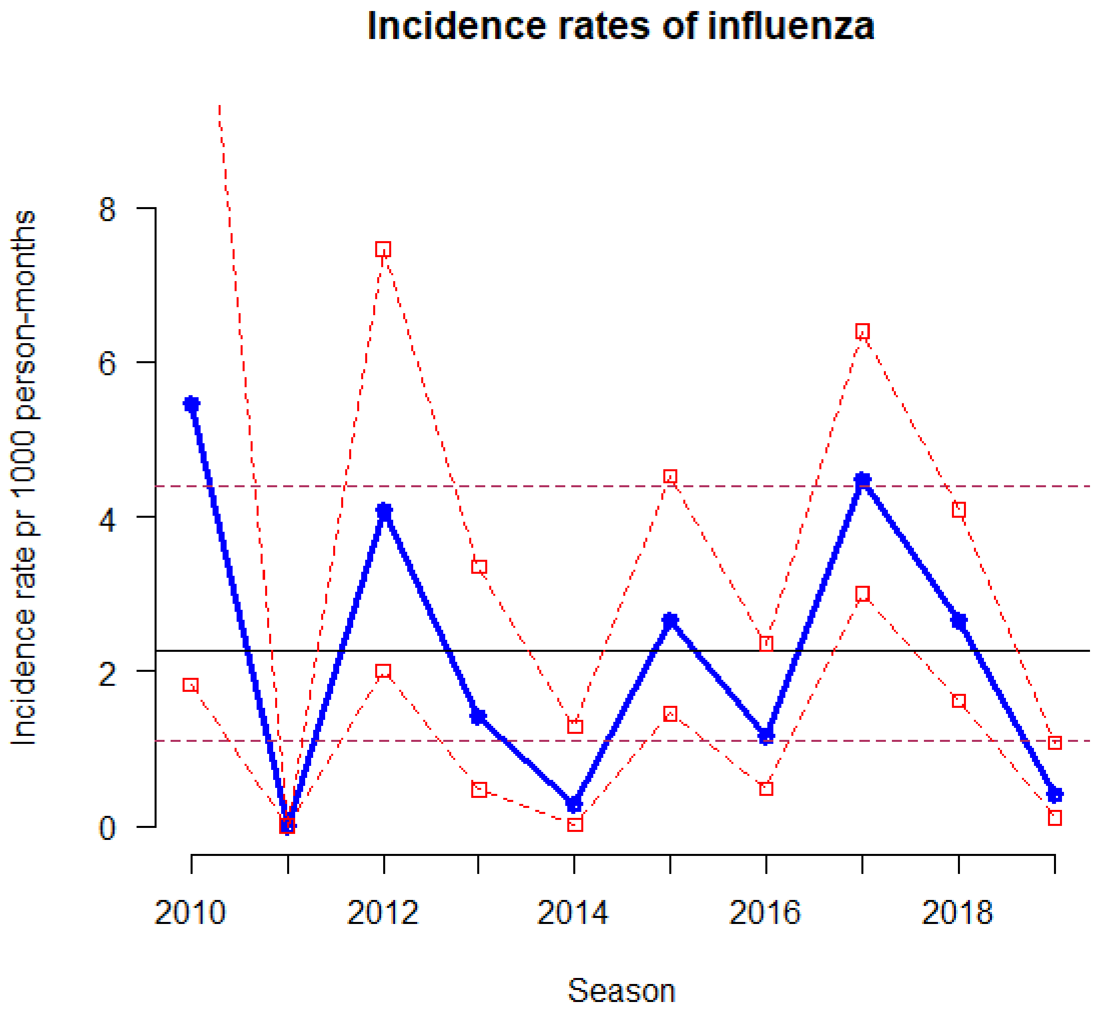

Supplementary Figure 1 Blue line: Incidence rate. Black line: average incidence rate across all seasons. Red lines: 95% CI. Maroon lines: average 95% CI.

**Supplementary Table 1: Influenza vaccinations in Denmark** (12)	
**Season**	**Strains in vaccination**
**2010/2011**	A/California/7/2009 (H1N1 like virus)<br>A/Perth/16/2009 (H3N2 like virus)<br>B/Brisbane/60/2008-like virus
**2011/2012**	A/California/7/2009 (H1N1 like virus)<br>A/Perth/16/2009 (H3N2 like virus)<br>B/Brisbane/60/2008-like virus
**2012/2013**	A/California/7/2009 (H1N1 like virus)

	A/Victoria/361/2011 (H3N2 like virus)<br>B/Wisconsin/1/2010-like virus
**2013/2014**	A/California/7/2009 (H1N1 like virus)<br>A/Victoria/361/2011 (H3N2 like virus)<br>B/Massachusetts/2/2012-like virus (Yamagata-line)
**2014/2015**	A/California/7/2009 H1N1 like virus<br>A/Texas/50/ 2012 H3N2 like virus<br>B/Massachusetts/2/2012-like virus (Yamagata-line)
**2015/2016**	A/California/7/2009/ H1N1 like virus<br>A/Switzerland/9715293/2013/H3N2-like virus<br>B/Phuket/3073/2013-like virus (Yamagata-line)
**2016/2017**	A/California/7/2009/ H1N1 like virus<br>B/Hong Kong/4801/2014 H3N2-like virus<br>B/Brisbane/60/2008-like virus (Victoria-line)
**2017/2018**	A/Michigan/45/2015 H1N1<br>B/Hong Kong/4801/2014 H3N2-like virus<br>B/Brisbane/60/2008-like virus (Victoria-line)
**2018/2019***	A/Michigan/45/2015 H1N1<br>A/Singapore/INFIMH-16_0019/2016 H3N2-like virus<br>B/Colorado/06/2017-like virus (Victoria-line)<br>Extra in the quadrivalent vaccine: B/Phuket/3073/2013-like virus (Yamagata-line)
**2019/2020***	A/Brisbane/02/2018 H1N1<br>A/Kansas/14/2017 H3N2-like virus<br>B/Colorado/06/2017-like virus (Victoria-line)<br>B/Phuket/3073/2013-like virus (Yamagata-line)

*until season 2017/2018 all influenza vaccines in Denmark contained 3 strains. In season 2018/2019 1/7 of the vaccines had four strains and in season 2019/2020 all vaccines were quadrivalent

Supplementary table 2: Incidence rates						
**Season**	**Number of patients in season, n**	**Follow-up in season, year**	**Influenza events in season, n**	**Incidence rate per 1000 person months (95%CI)**	**Incidence rate per 1000 person months in patients with same-season influenza vaccination (95%CI)**	**Incidence rate per 1000 person months in patients without same-season influenza vaccination (95%CI)**
**2010**	142	60.7	4	5.4 (1.8-12.9)	0 (0.0-19.5)	6.6 (2.2-15.7)
**2011**	262	129.9	0	0 (0.0-1.6)	0 (0.0-8.0)	0 (0.0-1.9)
**2012**	344	181.5	9	4.1 (2.0-7.4)	4.0 (0.8-12.9)	4.1 (1.8-8.1)
**2013**	451	233.7	4	1.4 (0.47-3.3)	1.3 (0.1-6.0)	1.5 (0.4-3.9)
**2014**	560	299.3	1	0.3 (0.02-1.3)	0 (0.0-3.0)	0.4 (0.0-1.7)
**2015**	683	370.7	12	2.7 (1.5-4.5)	1.8 (0.4-5.7)	3.0 (1.5-5.3)
**2016**	791	431.5	6	1.1 (0.48-2.4)	0.6 (0.1-2.8)	1.4 (0.5-3.1)
**2017**	912	497.9	27	4.5 (3.0-6.4)	4.1 (1.9-7.7)	4.6 (2.9-7.1)
**2018**	1012	558,1	18	2.6 (1.6-4.1)	1.6 (0.6-3.9)	3.2 (1.8-5.2)
**2019**	1061	606.7	3	0.41 (0.11-1.1)	0.4 (0.0-1.8)	0.4 (0.1-1.3)

Supplementary Table 3: Symptoms in relation to influenza						
**Transplanted organ**	**Influenza type**	**Symptoms in relation to influenza**	**Pneumonia**	**Hospital**	**ICU**	**Ventilator**
Liver	A	Fever, muscle pain	No	Yes	No	No
Liver	B	Fever, diarrhea, vomiting	No	Yes	No	No
Liver	B	Bronchitis symptoms	No	No	No	No
Liver	B	Fever	No	Yes	No	No
Liver	B	Fever, vomiting, muscle pain, shortness of breath	No	Yes	No	No
Liver	A	Fever, muscle pain	No	Yes	No	No

Liver	B	Fever	No	No	No	No
Liver	A	Fever, cough, headache, muscle pain	No	No	No	No
Liver	A	Pneumonia	Yes	Yes	Yes	Yes
Liver	B	Shortness of breath, muscle pain, headache, vomiting	No	No	No	No
Liver	A	Cough, throat pain	No	No	No	No
Liver	A	Fever, cough	No	Yes	No	No
Liver	B	Fever, abdominal pain, headache, cough	No	No	No	No
Liver	B	Unknown	No	No	No	No
Liver	A	Fever	No	Yes	No	No
Liver	A	Fever, headache, cough	No	Yes	No	No
Liver	B	Fever, headache, cough, joint pain	No	Yes	No	No
Liver	A	Fever, cough, muscle pain	No	No	No	No
Kidney	A	Fever, vomiting, cough	No	Yes	No	No
Kidney	B	Fever, cough	No	Yes	No	No
Kidney	A	Fever, cough	No	No	No	No
Kidney	A	Fever, cough, muscle- and joint pain	No	Yes	No	No
Kidney	A	Fever, nausea	No	Yes	No	No
Kidney	B	Fever, cough	No	Yes	No	No
Kidney	B	Pneumonia	Yes	Yes	No	No
Kidney	A	Decline in graft function, generally uncomfortable	No	Yes	No	No
Kidney	A	Fever, cough	No	Yes	No	No
Kidney	A	Fever, cough	No	Yes	No	No
Kidney	B	Headache, cough	No	Yes	No	No
Kidney	A	Pneumonia	Yes	Yes	No	No
Kidney	B	Tired	No	No	No	No
Kidney	A	Fever	No	Yes	No	No
Kidney	A	Unspecified influenza symptoms	No	No	No	No
Kidney	B	Fever, cough	No	Yes	No	No
Kidney	B	Vomiting, diarrhea, throat pain, respiratory insufficient	No	Yes	Yes	Yes
Kidney	A	Fever, cough	No	No	No	No
Kidney	B	Fever, cough	No	No	No	No
Kidney	A	Fever, cough, muscle- and joint pain, headache	No	Yes	No	No

Kidney	A	Pneumonia	Yes	Yes	No	No
Kidney	B	Fever, cough	No	Yes	No	No
Kidney	A	Fever	No	No	No	No
Kidney	A	Fever, respiratory insufficient	No	Yes	No	No
Kidney	A	Fever, cough, headache, tired	No	Yes	No	No
Kidney	A	Fever, respiratory insufficient	No	Yes	Yes	Yes
Kidney	B	Fever, cough, respiratory insufficient	No	Yes	No	No
Kidney	A	Fever, cough, joint pain	No	Yes	No	No
Kidney	A	Fever, muscle pain, cough	No	Yes	No	No
Kidney	A	Cough, respiratory insufficient	No	No	No	No
Kidney	B	Fever, nausea	No	No	No	No
Kidney	B	Pneumonia	Yes	Yes	No	No
Kidney	B	Fever, cough	No	No	No	No
Kidney	B	Pneumonia	Yes	Yes	Yes	Yes
Kidney	A	Pneumonia	Yes	Yes	No	No
Kidney	A	Fever, cough, nausea, headache	No	No	No	No
Kidney	B	Pneumonia	Yes	No	No	No
Kidney	A	Fever, cough	No	No	No	No
Kidney	B	Fever, cough, headache	No	No	No	No
Kidney	A	Asymptomatic	No	Yes	No	No
Kidney	A	Pneumonia	Yes	Yes	No	No
Kidney	A	Fever, vomiting, cough	No	Yes	No	No
Kidney	A	Dyspnea	No	Yes	No	No
Kidney	A	Fever, cough	No	No	No	No
Kidney	B	General discomfort	No	Unknown	No	No
Kidney	A	Fever, headache	No	Yes	No	No
Kidney	A	Pneumonia	Yes	Yes	No	No
Kidney	A	Unknown	No	No	No	No
Kidney	A	Cough	No	No	No	No
Kidney	A	Fever, headache, cough	No	Yes	No	No
Kidney	B	Fever, headache, muscle pain	No	No	No	No
Kidney	A	Pneumonia	Yes	Yes	No	No
Kidney	A	Pneumonia	Yes	No	No	No
Kidney	A	Fever, cough	No	No	No	No
Kidney	A	Fever, cough	No	Yes	No	No
Kidney	B	Sore throat	No	No	No	No

Kidney	A	Vomiting, cough	No	Yes	No	no
Kidney	A	Pneumonia	Yes	Yes	Yes	Yes
Kidney	A	Pneumonia	Yes	Yes	Yes	No
Kidney	A	Fever, cough	No	Yes	No	No
Kidney	A	Cough, abdominal pain, nausea, vomiting	No	Yes	No	No
Kidney	A	Cough	No	No	No	No
Kidney	B	Fever, cough	No	Yes	No	No
Kidney	B	Fever, diarrhea,	No	Yes	No	No
Kidney	A	Fever, headache, sore throat	No	Yes	No	No
Kidney	A	Fever, headache, cough, sore throat	No	Yes	No	No

Supplementary Table 4: Outcomes after influenza infection in recipients with/without same-season influenza vaccination

	**All influenza infections (n = 84)**	**Influenza infections in same-season vaccinated recipients (n = 19 (22.6%))**	**Influenza infections in non-vaccinated recipients (n = 65 (77%)**	**P-values**
**Time from tx to influenza, days, median (IQ range)**	824 (251.3-1929.8)	762 (323-1938)	826 (229-1928)	P=1
**Treated with oseltamivir (%)**	55 (65.5%)	15 (78.9%)	40 (61.5%)	P=0.6
**Pneumonia (%)**	14 (16.7%)	2 (10.5%)	12 (18.5%)	P=0.7
**Hospital admission (%)**	55 (65.5%)	14 (73.7%)	41 (63.1%)	P=0.8
**ICU admission (%)**	6 (7.1%)	1 (5.3%)	5 (7.7%)	P=1
**Mechanical ventilation (%)**	5 (6.0%)	1 (5.3%)	4 (6.2%)	P=1
**Death, 30 days all-cause mortality (%)**	1 (1.2%)	0 (0%)	1 (1.5%)	P=1
**Influenza type (%)**				
- **A**	53 (63.1%)	10 (52.6%)	43 (66.2%)	P=0.7
- **B**	31 (36.9%)	9 (47.4%)	22 (33.4%)	P=0.7

**Supplementary Table 5: Outcome sorted by type of influenza**				
	**All**	**Influenza A**	**Influenza B**	**P-values**
**Number of patients in group, n**	84	53	31	
**Pneumonia, n (%)**	14	10 (18.9)	4 (12.9)	P=0.56
**Hospital admission, n (%)**	55	39 (73.6)	16 (51.6)	P=0.07
**ICU Admission, n (%)**	6	4 (7.5)	2 (6.5)	P=1
**Mechanical ventilation, n (%)**	5	3 (5.7)	2 (6.5)	P=1
